# Supplementary material for: Determinants of Kangaroo Mother Care among low-birth-weight infants in low resource settings
Source: PLOS Glob Public Health. 2023 Sep 12;3(9):e0002015. doi: 10.1371/journal.pgph.0002015 (PMC10497168; doi:10.1371/journal.pgph.0002015)
Supplement: Attachment — Submitted filename: Determinants of KMC.docx [file pgph.0002015.s001.docx]

This is a nice secondary analysis of an existing survey addressing important issues towards KMC.

The literature review:
Please include KMC decreases sepsis.

The methodology and results are well described.

The different models are confusing and not sure if it adds additional value to the results section. The final model is probably the most important. 

The discussion requires some reworking.

Please be consistent LBW < 2500g
Line 274; doesn't fit in the paragraph. Please remove
The discussion is all over the place and has lots of repetition.
I suggest dividing the discussion into:
Country level
Individual level 
Community level
Then discuss a few important findings under each section.
Don’t repeat in each paragraph that KMC uptake is low, rather address why it is low and how can the uptake be improved. Some of the issues are appropriately mentioned.
At a country level, why is KMC uptake low? Are all the determinants just pooled, put on a hierarchy level, and not individualized to each country?
Several statements in the discussion require references.

Line 359; should be a new subheading on the limitations and strengths of the study.
