# Supplementary material for: Determinants of Kangaroo Mother Care among low-birth-weight infants in low resource settings
Source: PLOS Glob Public Health. 2023 Sep 12;3(9):e0002015. doi: 10.1371/journal.pgph.0002015 (PMC10497168; doi:10.1371/journal.pgph.0002015)
Supplement: Attachment — Submitted filename: Response to reviewers.docx [file pgph.0002015.s002.docx]

The Editor,

PLOS GLOBAL PUBLIC HEALTH

Manuscript ID: PGPH-D-23-00773

Title: Determinants of Kangaroo Mother Care among Low-Birth-Weight Infants in Low Resource Settings

This is a nice secondary analysis of an existing survey addressing important issues towards KMC.

The literature review:
Please include KMC decreases sepsis.

Response: The authors appreciate this, and have included (Ellen et al., 2016) as a reference for KMC decreases sepsis.

The methodology and results are well described.

Response: The authors appreciate this feedback

The different models are confusing and not sure if it adds additional value to the results section. The final model is probably the most important.

Response: The authors understand and appreciate this perspective, but the authors still think that the models are a way of validating that the addition of the model selection criterion which adds weight to the reason for the use of a three-level hierarchical model.

The discussion requires some reworking.

Please be consistent LBW < 2500g

Response: The authors apologize for the error and have corrected this in the “dependent variable” subsection of the methods section. We have decided to proceed with <2.5kg, as against <2500g.

Line 274; doesn't fit in the paragraph. Please remove

Response: The authors have removed this from the discussion section.

The discussion is all over the place and has lots of repetition.
I suggest dividing the discussion into:
Country level
Individual level 
Community level
Then discuss a few important findings under each section.
Response: The authors appreciate this comment and agree that this would make the discussion easier to read. We have reworked the discussion to fit this suggestion.

Don’t repeat in each paragraph that KMC uptake is low, rather address why it is low and how can the uptake be improved. Some of the issues are appropriately mentioned.
At a country level, why is KMC uptake low? Are all the determinants just pooled, put on a hierarchy level, and not individualized to each country?

Response: The authors have addressed this issue, to the best of our ability. Determinants were pooled and put on a hierarchical level and was not individualized to each country. This is because it would take a lot more than one manuscript to individualize these results to each country. The hierarchical modelling approach requires that determinants be pooled but with a nested structure to accommodate community and country level characteristics.

Several statements in the discussion require references.

Response: The authors have added references to required statements. We appreciate the constructive feedback to make the paper better.

Line 359; should be a new subheading on the limitations and strengths of the study.

Response: The authors appreciate this and have incorporated it into the reviewed manuscript

Lawal Temitayo

For the authors
